# Optimal Performance of Thin-Film Composite Nanofiltration-Like Forward Osmosis Membranes Set Off by Changing the Chemical Structure of Diamine Reacted with Trimesoyl Chloride through Interfacial Polymerization

**DOI:** 10.3390/polym13040544

**Published:** 2021-02-12

**Authors:** Manuel Reyes De Guzman, Micah Belle Marie Yap Ang, Shu-Hsien Huang, Qing-Yi Huang, Yu-Hsuan Chiao, Kueir-Rarn Lee

**Affiliations:** 1 Material Corrosion and Protection Key Laboratory of Sichuan Province, School of Materials Science and Engineering, Sichuan University of Science and Engineering, Zigong 643000, China; manuelrdg@yahoo.com; 2 R&D Center for Membrane Technology and Department of Chemical Engineering, Chung Yuan Christian University, Taoyuan 32023, Taiwan; mbmyang@gmail.com (M.B.M.Y.A.); krlee@cycu.edu.tw (K.-R.L.); 3 Department of Chemical and Materials Engineering, National Ilan University, Yilan 26047, Taiwan; jordan20310687@gmail.com; 4 Department of Chemical Engineering, University of Arkansas, Fayetteville, AR 72701, USA; ychiao@uark.edu; 5 Research Center for Circular Economy, Chung Yuan Christian University, Taoyuan 32023, Taiwan

**Keywords:** thin-film composite membranes, forward osmosis, interfacial polymerization, polyamide, membrane separation

## Abstract

Thin-film composite (TFC) polyamide membranes formed through interfacial polymerization can function more efficiently by tuning the chemical structure of participating monomers. Accordingly, three kinds of diamine monomers were considered to take part in interfacial polymerization. Each diamine was reacted with trimesoyl chloride (TMC) to manufacture TFC polyamide nanofiltration (NF)-like forward osmosis (FO) membranes. The diamines differed in chemical structure; the functional group present between the terminal amines was classified as follows: aliphatic group of 1,3-diaminopropane (DAPE); cyclohexane in 1,3-cyclohexanediamine (CHDA); and aromatic or benzene ring in m-phenylenediamine (MPD). For FO tests, deionized water and 1 M aqueous sodium sulfate solution were used as feed and draw solution, respectively. Interfacial polymerization conditions were also varied: concentrations of water and oil phases, time of contact between the water-phase solution and the membrane substrate, and polymerization reaction time. The resultant membranes were characterized using attenuated total reflectance-Fourier transform infrared spectroscopy, field emission scanning electron microscopy, atomic force microscopy, and surface contact angle measurement to identify the chemical structure, morphology, roughness, and hydrophilicity of the polyamide layer, respectively. The results of FO experiments revealed that among the three diamine monomers, CHDA turned out to be the most effective, as it led to the production of TFC NF-like FO membrane with optimal performance. Then, the following optimum conditions were established for the CHDA-based membrane: contact between 2.5 wt.% aqueous CHDA solution and polysulfone (PSf) substrate for 2 min, and polymerization reaction between 1 wt.% TMC solution and 2.5 wt.% CHDA solution for 30 s. The composite CHDA-TMC/PSf membrane delivered a water flux (*Jw*) of 18.24 ± 1.33 LMH and a reverse salt flux (*Js*) of 5.75 ± 1.12 gMH; therefore, *Js*/*Jw* was evaluated to be 0.32 ± 0.07 (g/L).

## 1. Introduction

Technologies continually develop for the sake of industrialization and the growing population. New technologies entail processing and fabrication of materials, which require intensive use of water and other solvents. Accordingly, the extent of pollution worsens or the level of environmental contaminants, produced as by-products of such processes, rises. For this reason, governments enjoin industries to build their own wastewater treatment facilities or to discharge their effluents into wastewater treatment plants. Traditional methods of wastewater treatment exist. But membrane separation processes have more compact systems, incur lower costs, and are more efficient [1].

Membrane technologies have been instrumental in solving problems on scarcity of drinking water and water for daily use [2,3,4]. Forward osmosis (FO), one of the membrane processes, attracts research interests because it can produce energy and clean water simultaneously [5,6]. FO is an osmotically-driven process that consumes less energy compared with the other osmotically-driven or filtration processes such as ultrafiltration, nanofiltration, and reverse osmosis. Studies have demonstrated that FO can be applied not only to the water sector but also to energy systems and the life sciences [7].

Hydration Technology Inc. developed the first commercial FO membranes fabricated from cellulose triacetate [8]. However, the membrane efficiency was insufficient to meet the large quantity of water needed by increasing consumers [9]. Fabricating efficient membranes depend largely on the nature of composite materials, which can be made from a combination of several polymers or from a mixture of polymers and inorganic additives or fillers [10]. A number of researchers [11,12,13,14] have put forth efforts to explore thin-film composite (TFC) membranes for FO processes. An advantage of TFC membranes rests on the possibility of optimizing each layer of composite membranes [15,16,17]. Moreover, the top selective layer is very thin and dense and, thus, provides high flux and high salt rejection.

FO membranes may have selective layers that are similar to those of reverse osmosis (RO) membranes. Commonly, the layer is composed of polyamide. Furthermore, the selective layers of FO membranes may also resemble those of nanofiltration (NF) membranes. Many researchers [18,19,20] have developed FO membranes that perform like NF membranes, as if they had NF selective layers. Hence, RO-like or NF-like selective layers can be used for FO processes, depending on the target application. For example, NF-like FO membranes usually find application in treating wastewater comprising a lot of multivalent ions. In this case, these NF-like FO membranes are more efficient than RO-like FO membranes.

Generally, TFC FO membranes are fabricated through interfacial polymerization, where diamines react with acyl chlorides to form a selective polyamide layer. Tuning the properties of either of these two monomers can enhance the selective layers of TFC FO membranes. Accordingly, membranes with superior efficiency can be produced if suitable monomers are chosen [21,22,23], compatible supporting layers are used [24,25], and additives are incorporated [26,27].

In this work, three types of diamine monomers were explored to fabricate TFC NF-like FO membranes—1,3-diaminopropane (DAPE), 1,3-cyclohexanediamine (CHDA), and m-phenylenediamine (MPDA). Although these monomers were all diamines, their structures differed in terms of the moiety in between the terminal amines. In that regard, we considered such types of diamine monomers to investigate if they could be potential candidates to replace MPD, a commonly used monomer for fabricating RO or FO membranes, with the goal of fabricating FO membranes with higher efficiency. DAPE consisted of an aliphatic group in the middle of two terminal amines. CHDA had a cycloaliphatic group between the amines, whereas MPDA contained an aromatic ring. In interfacial polymerization, the diamine monomer structure affects its reactivity with the acyl chloride group. In turn, differing reactivities will bring about changes in the final morphology, hydrophilicity, and degree of cross-linking. Variation in such properties would influence the performance of various kinds of membranes in different ways. In this context, the dense pervaporation membranes we prepared in our previous study [28] differed in characteristics and efficiencies from the FO membranes in this present study, despite the same diamine monomers were employed. Furthermore, the optimization procedures depended largely on the type of membrane and its fabrication, and not on the similarity of monomers used in the process.

## 2. Methods

### 2.1. Materials

Three kinds of diamine monomers were used, namely DAPE, CHDA, and MPDA. Each of them was reacted with trimesoyl chloride (TMC), an acyl chloride monomer, through interfacial polymerization. DAPE, CHDA, and TMC were obtained from Tokyo Chemical Industry Co. Ltd. (Tokyo, Japan), whereas MPDA was from Merck & Co., Kenilworth, NJ, USA. The membrane support was made of polysulfone or PSf (UDEL P-3500), which was supplied by Amoco Performance Product Inc., Ridgefield, CT, USA. Polyethylene glycol 20 k (PEG20k) was provided by Alfa Aesar (Heysham, Lacashire, England). 1-Methyl-2-pyrrolidinone (NMP) and n-hexane solvents were purchased from Tedia Company Inc. (Fairfield, OH, USA). Sodium sulfate (Na_2_SO_4_) was delivered by Nihon Shiyaku Industries Ltd. (Tokyo, Japan).

### 2.2. Fabrication of Composite Membranes

A solution of 16 wt.% PSf in NMP was prepared by dissolving PSf pellets in NMP. After the pellets were completely dissolved, PEG20k was introduced as an additive. The amount of PEG20k added was 50 wt.% of the amount of PSf. When PEG20k was completely dissolved, the solution was degassed by placing it in an ultrasonicator for 10 min. Then, the solution was cast on a glass plate wrapped with a nonwoven polyester. A 200-µm casting knife was used for that purpose. The cast film was precipitated into a membrane through a wet-phase inversion method in a coagulation bath containing distilled water. Afterward, the membrane was washed with distilled water several times to remove the excess NMP solvent. Finally, the membrane was stored in fresh distilled water before it was used for interfacial polymerization.

**Figure 1:**
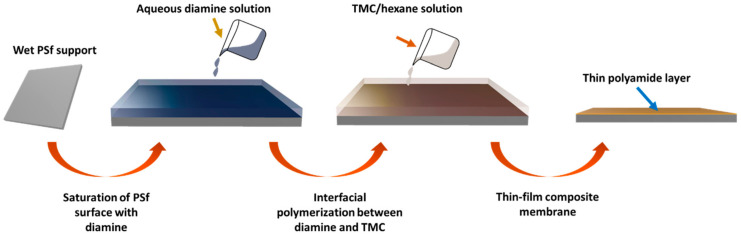
Fabrication of thin-film composite membrane through interfacial polymerization.

**Figure 2:**
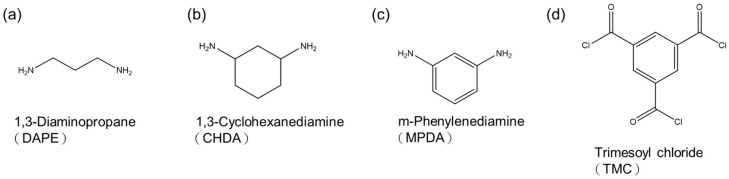
Chemical structure of monomers: (**a**) DAPE; (**b**) CHDA); (**c**) MPDA; (**d**) TMC.

Figure 1 illustrates the steps involved in interfacial polymerization to fabricate TFC polyamide membranes. The concentration of aqueous amine solution was 2 wt.%. The diamine monomer was either DAPE, CHDA, or MPDA. Each monomer differed in chemical structure, as shown in Figure 2, which also includes the structure of TMC. Then, an organic phase was prepared, a solution of TMC in n-hexane, with a concentration of 1 wt.%. First, a PSf membrane support, which was previously stored in distilled water, was pressed gently with a rubber roller to remove the excess water. It was then clamped onto an iron stage. Afterward, the aqueous amine solution was poured onto the surface of PSf, allowing the solution to reside there for 2 min. After that, the solution was poured off. To remove the excess amine solution, the wet PSf was gently pressed with a rubber roller. Subsequently, the TMC solution was poured onto the surface of the wet PSf, allowing it to get in contact with the sorbed amine in PSf for 30 s. At that point, an instantaneous reaction between the amine and TMC ensued, forming a thin film or layer on the PSf support. Finally, the membrane was washed with distilled water; after washing, it was stored in fresh distilled water.

### 2.3. Characterization

To generate data on surface structure, to be analyzed for chemical or functional groups present, attenuated total reflectance-Fourier transform infrared (ATR-FTIR) spectroscopy (Perkin Elmer Spectrum 100 FTIR Spectrometer, Waltham, MA, USA) was used. As to the analysis of surface elemental compositions, X-ray photoelectron spectroscopy (XPS, VG K-alpha ThermoFisher Scientific, Inc., Waltham, MA, USA) was employed. Differences in surface morphology were determined using field emission scanning electron microscopy (FESEM, S-4800, Hitachi Co., Tokyo, Japan). Surface roughness was evaluated using atomic force microscopy (AFM, FSM Nanoview 1000–2000, Labtrek S.r.l, Padova, Italy). The wettability of membranes in water was measured using an automatic interfacial tensiometer (PD-VP Model, Kyowa Interface Science Co. Ltd., Niiza-City, Saitama, Japan).

### 2.4. Forward Osmosis Experiments

Similar setup and procedures, described in our previous work [11,12], were used to determine the membrane performance. The membrane selective layer faced the feed solution (deionized water), and it had an effective area of 12.24 sq. cm. As to the draw solution, 1 M Na_2_SO_4_ was employed. The flux (*J_w_*) was calculated as follows:

(1) $$J_{w}=\frac{\Delta V}{A\Delta t}$$

where Δ*V*, *A*, and Δ*t* were the change in volume, effective membrane surface area, and change in time, respectively. The reverse solute flux (*J_s_*) was calculated as follows:

(2) $$J_{s}=\frac{\Delta \left(C_{t}V_{t}\right)}{A\Delta t}\;$$

where *C_t_* and *V_t_* represented the salt concentration and feed volume, respectively.

## 3. Results and Discussion

### 3.1. Chemical Structure

**Figure 3:**
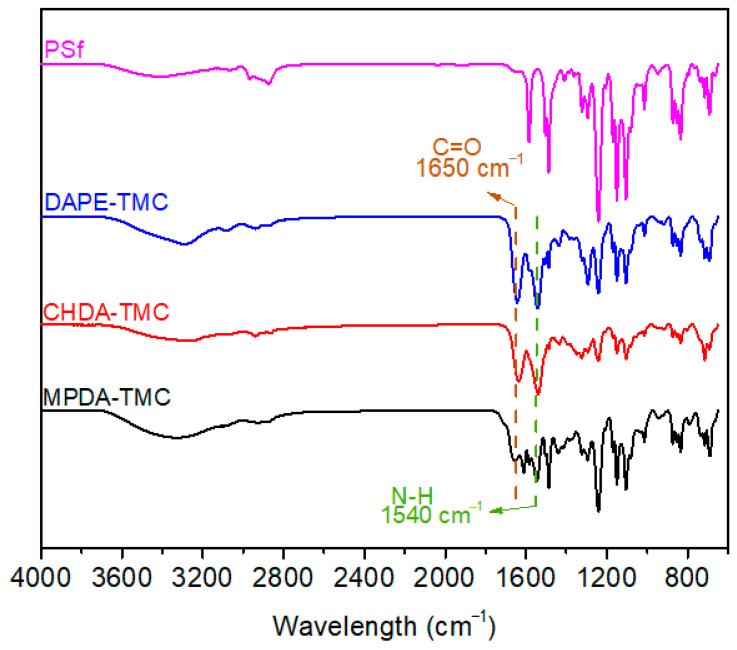
ATR-FTIR spectra of PSf support and TFC membranes.

Figure 3 represents ATR-FTIR spectra of PSf and TFC membranes. As a result of interfacial polymerization reactions between TMC and each of the three diamine monomers (DAPE, CHDA, MPDA), new peaks appeared at 1650 and 1540 cm^−1^. These two peaks referred to C=O (amide I) and NH (amide II) of polyamide, respectively [29,30]. MPDA-TMC had a peak at 1612 cm^−1^, corresponding to C=C of benzene ring in the structure of MPDA. The aforementioned results validated the formation of a polyamide layer on PSf.

**Figure 4:**
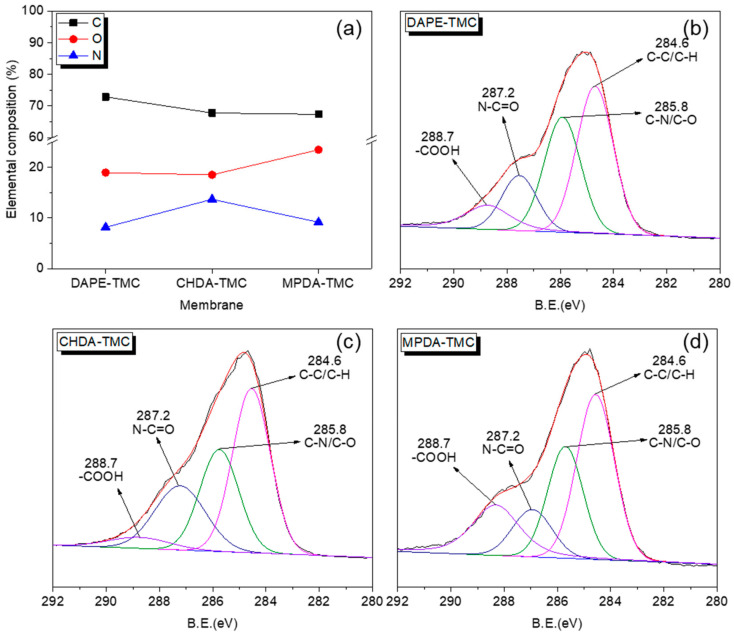
(**a**) Elemental compositions and (**b**–**d**) C1s spectra of TFC membranes.

Elemental and chemical compositions were obtained from XPS analysis (Table 1 and Figure 4). Figure 4a indicates that CHDA-TMC had the highest N content among the TFC membranes. This result suggested the presence of many cross-linked amide groups on the surface. Chemical compositions (Figure 4b–d) were based on C1s spectra from the XPS analysis.

Table 1 tabulates data derived from spectral analysis. The data confirmed that CHDA-TMC contained the highest percentage of amide groups (23.06% at 287.2 eV). On the other hand, MPDA-TMC showed the greatest carboxyl groups, amounting to 19.52%. This much carboxyl content might be attributed to the benzene ring in MPDA. DAPE consisted of an aliphatic group, whereas CHDA had cyclohexane. However, MPDA comprised a benzene ring, which was responsible for the greatest steric hindrance during interfacial polymerization. Such an effect decreased the degree of cross-linking between the amines and TMC.

### 3.2. Membrane Morphology and Hydrophilicity

**Figure 5:**
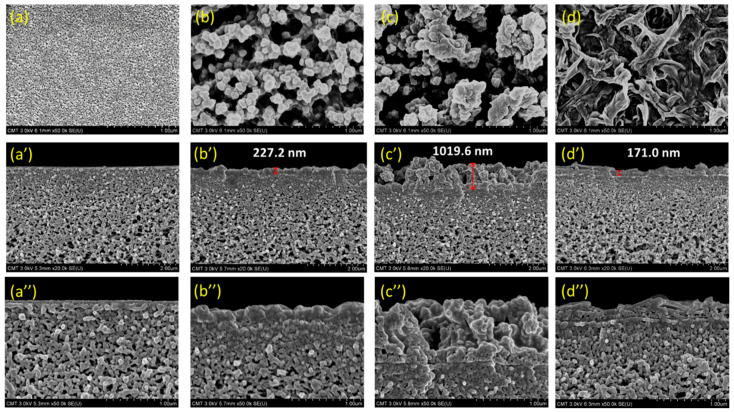
FESEM images: (**a**,**a**’,**a**’’) PSf support; (**b**,**b**’,**b**’’) DAPE-TMC; (**c**,**c**’,**c**’’) CHDA-TMC; (**d**,**d**’,**d**’’) MPDA-TMC.

Varying monomer chemical structures lead to different surface morphologies and roughness. The variation in structures affects the reaction rates. Figure 5 depicts FESEM images of PSf and TFC membranes. The PSf support displayed a smooth surface (Figure 5a), while TFC membranes described different surface morphologies (Figure 5b–d) because of the dissimilar structures of amines that reacted with TMC during interfacial polymerization. DAPE included an aliphatic group in its structure, whereas CHDA had a cycloaliphatic group, and these two monomers led to the production of nodules on the membrane surface. For the morphology of MPDA with an aromatic ring between its amines, the result was a surface with ridges and valleys [31].

Cross-sectional images in Figure 5b’,b’’–d’,d’’ (high-resolution or magnified images illustrated in Figure 5b’’–d’’) compare the thickness of selective layers in DAPE-TMC, CHDA-TMC, and MPDA-TMC. Among these composite membranes, CHDA-TMC exhibited the thickest layer, measuring 1019.6 ± 72.08 nm. Its selective layer was about 5 times thicker than that of DAPE-TMC (227.2 ± 11.76 nm), and about 6 times thicker than that of MPDA-TMC (171.0 ± 7.50 nm). CHDA-TMC turned out to have the thickest layer, probably because of the fast reaction between CHDA and TMC. Generally, the thickest polyamide layer would exhibit the highest degree of cross-linking. And this correlation was indeed the case herein, as indicated by the XPS analysis.

Because of steric hindrance caused by the benzene group in MPDA, the polyamide layer in MPDA-TMC acquired a low degree of cross-linking, and it came out to be the thinnest layer. Moreover, because of the aromatic ring in MPDA, electron orbitals of the π bond in the polymer chain overlapped and formed a π-π stacked arrangement. Accordingly, the benzene ring was in a stacked arrangement. Hence, the aromatic ring was not easily twisted and deformed, making it difficult for MPDA to penetrate the polyamide layer and diffuse toward TMC to initiate reaction.

**Figure 6:**
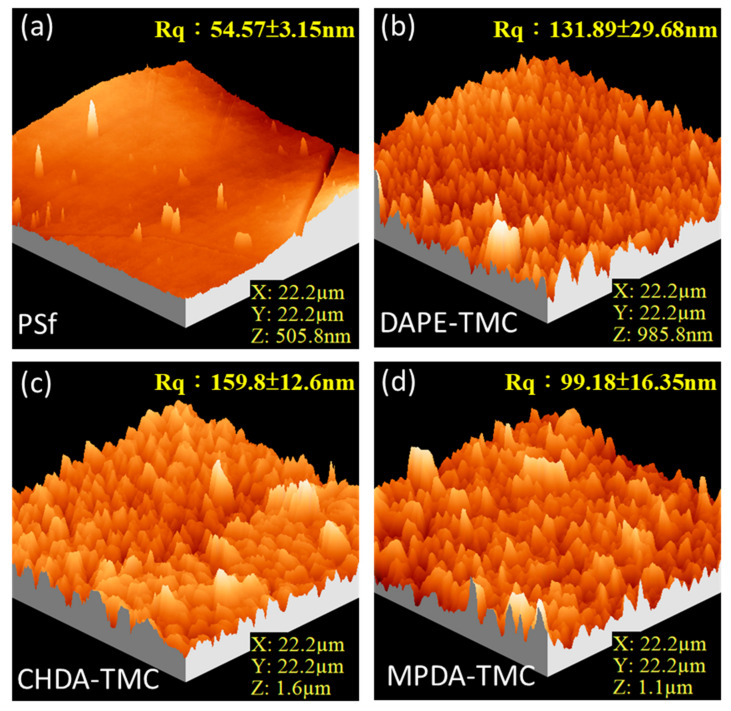
Three-dimensional AFM images: (**a**) PSf support; (**b**) DAPE-TMC; (**c**) CHDA-TMC; (**d**) MPDA-TMC.

Figure 6 delineates three-dimensional AFM images of the PSf support and TFC membranes. Pristine PSf exhibited the smoothest surface (54.57 ± 3.15 nm) (Figure 6a). However, TFC membranes were rough on the surface (Figure 6b–d). The roughness increased in this order: MPDA-TMC (99.18 ± 16.35 nm) < DAPE-TMC (131.89 ± 29.68 nm) < CHDA-TMC (159.8 ± 12.6 nm). These surface roughness results agree with the FESEM image analysis. The data on roughness implied the occurrence of fast reaction between CHDA and TMC, producing more nodules contributing to very high surface roughness.

**Figure 7:**
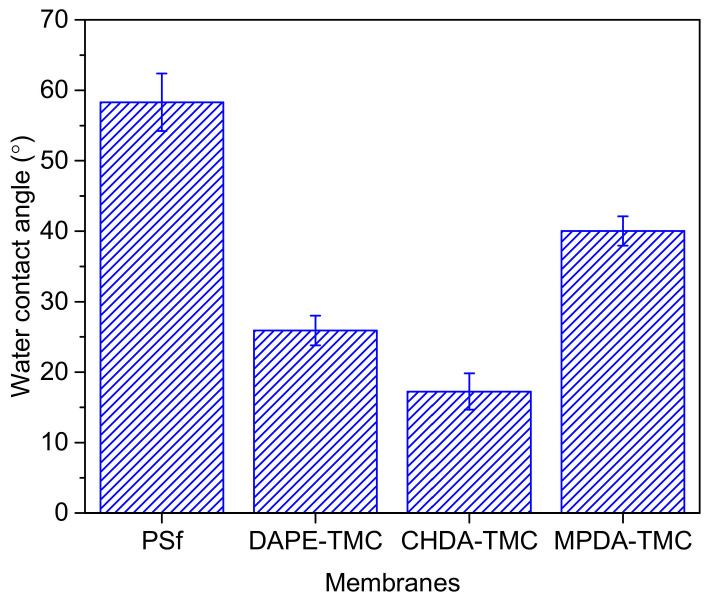
Hydrophilicity of PSf support and TFC membranes.

The membrane hydrophilicity is a function of the surface chemical functional groups or morphology. Figure 7 compares water contact angle data for PSf and TFC membranes. The PSf surface measured 58.31 ± 4.09° in contact angle, similar to that observed in our prior work [32]. The low contact angle for PSf might be ascribed to the addition of PEG20k, which entangled with the PSf chains. As a result, the membrane was more hydrophilic than the PSf support without PEG20k. The TFC membranes formed through interfacial polymerization had lower water contact angle than the neat PSf.

Data on water contact angle for TFC membranes gave an increasing trend: CHDA-TMC (17.25 ± 2.59°) < DAPE-TMC (25.91 ± 2.10°) < MPDA-TMC (40.03 ± 2.9°). The surface of MPDA-TMC was the least hydrophilic, because the structure of MPDA consisted of a hydrophobic benzene ring. MPDA-TMC also had the smoothest surface. Generally, a smooth and hydrophilic surface provides a higher water contact angle than a rough surface [33]. On the other hand, CHDA-TMC with the roughest surface exhibited the most hydrophilic surface. Herein, surface roughness appears to dominate surface hydrophilicity.

### 3.3. Performance of TFC Membranes

**Figure 8:**
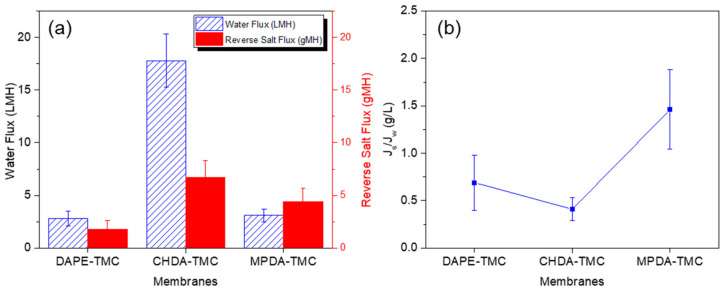
Comparison of (**a**) water flux and (**b**) reverse salt flux for various composite polyamide membranes. Parameters for FO tests: 1 M Na_2_SO_4_ draw solution and deionized water as feed solution.

Figure 8 describes the membrane performance in terms of water flux, reverse solute flux, and ratio of reverse solute flux to water flux. Data on water flux for TFC membranes (Figure 8a) followed this increasing order: DAPE-TMC (2.81 ± 0.73 LMH) < MPDA-TMC (3.1 ± 0.6 LMH) < CHDA-TMC (17.78 ± 2.54 LMH). A similar increasing order was observed for reverse solute flux (Figure 8a). CHDA-TMC delivered the highest water flux or reverse salt flux because it had the most hydrophilic surface. MPDA-TMC gave a higher water flux and reverse solute flux than DAPE-TMC. This behavior is probably because of the chemical structure of MPDA, consisting of a bulky benzene group that caused steric hindrance, leading to MPDA-TMC having a looser polyamide layer than DAPE-TMC, which contained an aliphatic group between its terminal amines. When this aliphatic group was polymerized with TMC to form polyamide, it packed together, producing a dense polyamide layer; hence, a low water flux was obtained.

The ratio of reverse solute flux to water flux dictates the efficiency of membranes subjected to FO tests. A lower ratio signifies higher efficiency. Figure 8b indicates that CHDA-TMC led to the lowest ratio of reverse solute flux to water flux, indicating it performed the best among the TFC membranes during the FO tests, with 1 M Na_2_SO_4_ as draw solution and deionized water as feed solution. In the following discussion, interfacial polymerization conditions for CHDA and TMC were further investigated to determine the optimum conditions that would result in high performance of FO membranes.

**Figure 9:**
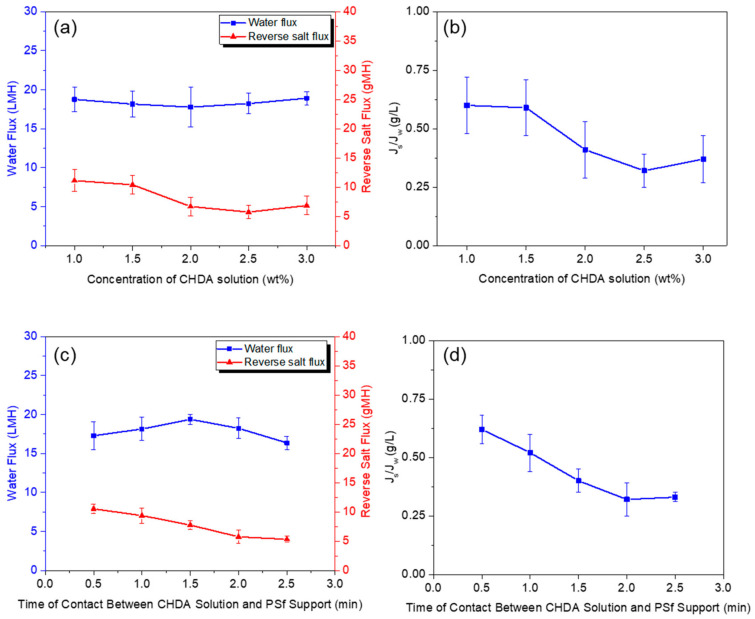
Effect of (**a**,**b**) aqueous CHDA concentration and (**c**,**d**) time of contact between CHDA solution and PSf support on water flux and reverse salt flux produced by composite polyamide membranes. (Polymerization conditions: 2-min contact between aqueous CHDA solution and PSf support; 30-s contact between 1 wt.% TMC solution and CHDA solution sorbed in PSf support.)

Figure 9 plots the effect of the following factors on the membrane performance: concentration of aqueous CHDA solution and its time of contact with the PSf support. As the CHDA concentration increased, the water flux remained similar, about 19 LMH (Figure 9a). On the other hand, the reverse salt flux decreased when the CHDA concentration was more than 2 wt.%, a result supporting that the membrane became denser, thereby less salt solution passed through the membrane.

As can be deduced from *Js*/*Jw* data (Figure 9b), 2.5 wt.% proved to be the optimum CHDA concentration required to make a high-performance FO membrane. When the time of contact between the aqueous CHDA solution and the PSf support was varied from 0 to 2.5 min (Figure 9c,d), the amine sorbed in the support differed in amount, and these changes affected the membrane performance. When the contact time was set to 1.5 min, the highest water flux was 19.39 ± 0.65 LMH. However, the lowest reverse salt flux (5.5 gMH) was recorded when the time was longer than 2 min.

In summary, the best-performing CHDA-TMC membrane was produced when the time of contact between the amine and the PSf support was 2 min, and the optimum concentration of aqueous CHDA solution was 2.5 wt.%.

**Figure 10:**
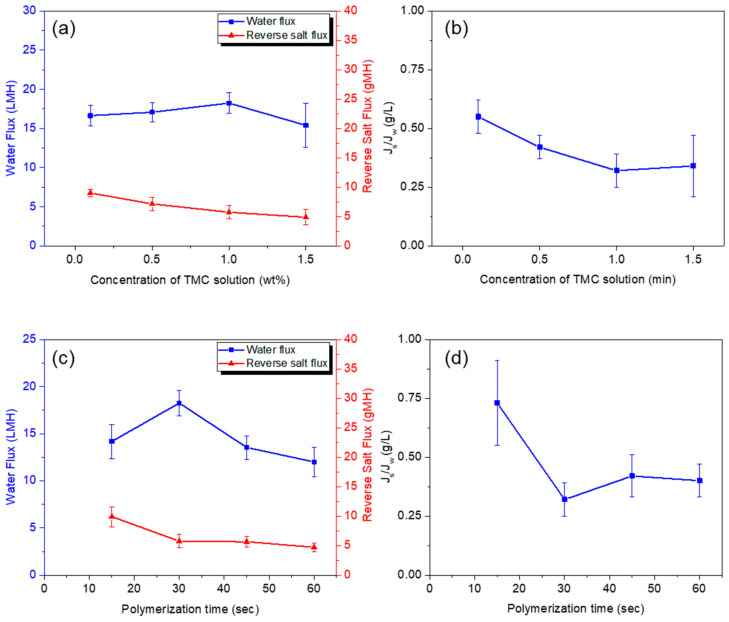
Effect of (**a**,**b**) TMC concentration and (**c**,**d**) polymerization time on water flux and reverse salt flux delivered by composite polyamide membranes. (Polymerization conditions: 2-min contact between PSf support and 2.5 wt.% aqueous CHDA solution; 30-s contact of TMC solution with CHDA solution sorbed in PSf support).

Figure 10 provides the performance of membranes fabricated at varied conditions of interfacial polymerization: concentration of TMC/n-hexane solution (Figure 10a,b) and time of polymerization reaction between TMC and CHDA (Figure 10c,d). When the concentration was raised from 0.1 to 1 wt.% TMC, the water flux increased from 16.64 to 18.24 LMH (Figure 10a). The reason was the high concentration of TMC; more acyl chloride groups derived more chances of hydrolyzing into COOH, which rendered the surface more hydrophilic (hence, enhanced water flux) [34]. However, when the concentration was further varied from 1 to 1.5 wt.%, the membrane became dense, as the water flux decreased to 15.4 LMH (Figure 10a). Furthermore, the reverse salt flux also went down to 4.89 gMH (Figure 10a), likewise an evidence of a dense membrane. According to the data on *Js*/*Jw* ratio (Figure 10b), the optimum TMC concentration was 1 wt.%.

When the polymerization reaction time was increased from 15 to 30 s, the water flux increased as well from 14.17 to 18.24 LMH (Figure 10c). When the time was longer, more acyl chloride groups hydrolyzed into COOH retained on the membrane surface. As such, the membrane was more hydrophilic; thus, the water flux was higher. However, the membrane became denser at time longer than 30 s. Accordingly, the water flux dropped to 12 LMH when the time reached 60 s (Figure 10c). On the other hand, the reverse salt flux plateaued at around 5 gMH by the time the polymerization reaction lasted from 30 to 60 s (Figure 10d). That means the membrane reached its densest form, wherein salts were prevented from being transmitted to the other side of the membrane. Based on the results of data on *Js*/*Jw* ratio (Figure 10d), the optimal polymerization reaction time was 30 s.

## 4. Conclusions

TFC NF-like FO membranes were successfully fabricated through interfacial polymerization wherein the chemical structure of diamine reacted with TMC was varied to determine which of the three diamines would lead to a composite membrane with optimal performance. That diamine monomer was identified to be CHDA, which contained a cycloaliphatic group between the terminal amines. Membrane characterizations and FO tests revealed the following optimum conditions: 2.5 wt.% aqueous CHDA solution; 2-min contact between CHDA solution and PSf support; 1 wt.% TMC solution; 30-s polymerization reaction between CHDA and TMC. The composite CHDA-TMC/PSf FO membrane turned out to have the most hydrophilic and roughest surface, and it delivered the best performance: *Jw* = 18.24 ± 1.33 LMH; *Js* = 5.75 ± 1.12 gMH; *Js*/*Jw* = 0.32 ± 0.07 (g/L).

## Figures and Tables

**Table 1:** Chemical functional groups on the surface of TFC membranes, as identified from XPS spectra.

Membranes	C–C/C–H (%)	C–N/C–O (%)	N–C=O (%)	–COOH (%)
DAPE-TMC	41.85	32.58	15.1	10.47
CHDA-TMC	43.98	28.2	23.06	4.76
MPDA-TMC	41.48	26.5	12.5	19.52

## Data Availability

Data is contained within the article.
